# Prevention and Management of Falls in Older Adults admitted to Woodlands Hospital, an inpatient Old Age Psychiatric Unit

**DOI:** 10.1192/j.eurpsy.2022.463

**Published:** 2022-09-01

**Authors:** N. Darod

**Affiliations:** GMMH, Bolton Old Age Cmht, Breightmet Health Centre, Bolton, Manchester, United Kingdom

**Keywords:** falls, falls prevention, old age psychiatry, falls management

## Abstract

**Introduction:**

Falls are a significant cause of injury in older adults who are disproportionately at higher risk due to multiple factors such as, mobility issues, co-morbidities and polypharmacy. There are several evidence-based interventions that can be implemented to reduce the risk of – and manage falls.

**Objectives:**

Assess whether Woodlands Hospital has implemented the standards set by NICE guidelines on the management of Falls in Older People.

**Methods:**

Retrospective audit of patients admitted to Woodlands Hospital from 1^st^ June to 1^st^ December 2018. 113 patient records were analysed to determine; was an falls risk assessment completed on admission, did patients ’at risk of falls’ have individualized interventions in place, was a falls risk assessment completed weekly at MDT, following a fall, were patients checked for signs of fracture before moved, was a medical examination completed and were neurological observations completed in patients with observed head injury or where it could not be excluded?

**Results:**

100% of patients had individualised interventions to reduce risk of falls and 97.3% of patients had an assessment of risks completed on admission. 60.3% of patients were checked for signs of fracture. 78.3% of patients had a physical examination within 12 hours. 75% of patients had neurological observations completed.

**Conclusions:**

Risk assessment for falls and individualized interventions for patients at risk of falls were completed at a high standard. There remains scope for improvement of review of risk of falls during weekly MDT, documentation of checking for signs of fractures and neurological examination. The outcomes were relayed to the unit and plans to re-audit in September 2021.

**Disclosure:**

No significant relationships.

